# Are our diets getting healthier and more sustainable? Insights from the European Prospective Investigation into Cancer and Nutrition – Netherlands (EPIC-NL) cohort

**DOI:** 10.1017/S1368980019001824

**Published:** 2019-07-31

**Authors:** Sander Biesbroek, WM Monique Verschuren, Jolanda MA Boer, Yvonne T van der Schouw, Ivonne Sluijs, Elisabeth HM Temme

**Affiliations:** 1 Centre for Nutrition, Prevention and Health Services, National Institute for Public Health and the Environment (RIVM), Antonie van Leeuwenhoeklaan 9, Bilthoven, 3721 MA The Netherlands; 2 Julius Center for Health Sciences and Primary Care, University Medical Centre Utrecht, Utrecht University, Utrecht, The Netherlands

**Keywords:** Diet, Nutritional quality, Environmental impact, 20 year, Dutch cohort

## Abstract

**Objective:**

To identify differences in dietary quality, dietary greenhouse gas (GHG) emissions and food consumption over 20 years in a Dutch cohort.

**Design:**

Participants (*n* 8932) filled out an FFQ in 1993–1997 and in 2015. The Dutch Healthy Diet index 2015 (DHD15-index) score, GHG emissions and consumption of food groups (g/4184 kJ (1000 kcal)) were compared between the time points with paired *t* tests.

**Setting:**

The Netherlands.

**Participants:**

European Prospective Investigation into Cancer and Nutrition – Netherlands (EPIC-NL) cohort, aged 18–65 years at baseline.

**Results:**

Total energy intake decreased by –678 (95 % CI –4908, 3377) kJ/d (–162 (95 % CI –1173, 807) kcal/d) for men and –372 (95 % CI –3820, 3130) kJ/d (–89 (95 % CI –913, 748) kcal/d) for women. DHD15-index scores increased by 11 % (from 64·8 to 71·9 points) and 13 % (from 65·2 to 73·6 points) in men and women, respectively (*P* < 0·0001), mainly due to an increased (shell)fish and nuts/seeds/nut paste consumption. After energy intake adjustment, dietary-related GHG emissions increased by 5 % in men (2·48–2·61 kg CO_2_-eq/4184 kJ (1000 kcal), *P* < 0·0001) and were similar in women (0·4 %, 2·70–2·71 kg CO_2_-eq/4184 kJ (1000 kcal), *P* = 0·3930) due to the increased consumption of (shell)fish, nuts/seeds/nut paste, poultry and higher GHG-intensive red meats such as beef.

**Conclusions:**

This Dutch cohort analyses showed more healthy diets without mitigated GHG emissions over a 20-year period, at similar energy intakes. Higher consumption of (shell)fish and poultry was not yet at the expense of red and processed meat. Lower consumption of animal-based foods is needed to achieve healthier as well as environmentally friendly diets.

Diet is a major contributor to both global warming and health. On a global scale, current food production is estimated to be responsible for 20–30 % of total greenhouse gas (GHG) emissions and thus has a major impact on the environment^(^ ^1^ ^–^ ^3^ ^)^. In addition, the global food system faces the major challenge producing food for the ever-growing world population while dealing with limited available resources^(^ ^4^ ^)^. The Paris Climate Agreement and the UN Sustainable Development Goals stress the urgency of implementing new strategies towards a more sustainable future^(^ ^5^ ^,^ ^6^ ^)^.

Both current consumer awareness of the environmental impact of the diet and the willingness to adapt to a new type of diet are proven to be low^(^ ^7^ ^,^ ^8^ ^)^. The current Western diet is considered environmentally unsustainable and unhealthy due to the high quantities of animal-based foods consumed^(^ ^9^ ^,^ ^10^ ^)^. Shifts in dietary patterns towards less animal- and more plant-based foods can therefore potentially provide benefits for both the environment and health, as has been described by previous review studies^(^ ^11^ ^–^ ^14^ ^)^.

In the last decades, the food production and consumption landscape in the Netherlands has changed considerably. The area of agricultural land decreased by 6 % between 2000 and 2011, while food production increased by 9 %^(^ ^15^ ^)^. In two representative Dutch samples several dietary trends were identified between 2007–2010 and 2012–2014, such as lower consumption of meat and potatoes but stable consumption of vegetables^(^ ^16^ ^)^.

Recognizing and understanding trends in food consumption is important to accurately design policy measures (such as information strategies, food-based dietary guidelines, taxes/subsidies on certain foods and other measures) aiming towards a combined environmentally sustainable and healthy food consumption and to evaluate their effectiveness. Since a changed food consumption pattern may affect the quality of the diet as well as its environmental impact, it is important to identify these trends. Therefore, our current study was set up to: (i) identify the differences in food consumption and dietary quality over the course of 20 years using the European Prospective Investigation into Cancer and Nutrition – Netherlands (EPIC-NL) cohort; and (ii) to calculate the environmental impact in terms of GHG emissions of the observed differences in both men and women.

## Participants and methods

### Study population

EPIC-NL^(^ ^17^ ^)^ consists of 40 011 participants of the two Dutch contributors to the European-wide EPIC project, being EPIC-Prospect^(^ ^18^ ^)^ and EPIC-MORGEN^(^ ^19^ ^,^ ^20^ ^)^, both carried out between 1993 and 1997. The EPIC-Prospect cohort included 17 357 women aged 49–70 years living in the city of Utrecht and its vicinity. The EPIC-MORGEN cohort included 22 654 men and women aged 20–65 years living in Amsterdam, Maastricht and Doetinchem. The design and rationale of EPIC-NL are described elsewhere by Beulens *et al.* ^(^ ^17^ ^)^.

In 2015, respondents to the 2011 follow-up questionnaire on electromagnetic radiation^(^ ^21^ ^)^ who were still alive, living in the Netherlands and who gave informed consent (*n* 13 421) were invited to fill out an FFQ. In addition, participants from Doetinchem who at that time had already participated in the sixth round of the Doetinchem cohort study (*n* 1528) were invited. Participants from Doetinchem did not receive the electromagnetic radiation questionnaire. The response rate to the 2015 FFQ was 62·9 % (9399 out of 14 949).

For the current study, participants without dietary information at baseline were excluded (*n* 27). Participants with implausible dietary intake at either FFQ, i.e. those with a reported energy intake of less than 2092 kJ/d (500 kcal/d) or greater than 14 644 kJ/d (3500 kcal/d)^(^ ^22^ ^)^, were also excluded (*n* 440) resulting in a total population of 8932 participants (see online supplementary material, Supplemental Fig. S1).

### Dietary assessment

At baseline, usual daily dietary intake was estimated by a 178-item FFQ, which has been validated against twelve 24 h dietary recalls and biomarkers in 24 h urine and blood^(^ ^23^ ^,^ ^24^ ^)^. Spearman rank correlation coefficients based on estimates of the FFQ and 24 h recalls were 0·58 for potatoes, 0·38 for vegetables, 0·68 for fruits, 0·47 for meat, 0·32 for fish, 0·64 for cheese, 0·71 for dairy, 0·78 for sweet products, and 0·56 for biscuits and pastry. Energy intake was estimated using the 1996 Dutch Food Composition Table^(^ ^25^ ^)^.

At follow-up, a new standardized 160-item FFQ developed for Dutch epidemiological studies was used^(^ ^26^ ^)^. This FFQ was validated against on average 2·7 (range 1–5) telephone-based 24 h recalls as well as biomarkers in 24 h urine and blood samples. Spearman rank correlation coefficients based on estimates of the FFQ and 24 h recalls were 0·28 for potatoes, 0·53 for vegetables, 0·67 for fruits, 0·38 for meat, 0·28 for fish, 0·16 for cheese, 0·61 for dairy, 0·38 for sweet products, and 0·33 for biscuits and pastry. Energy intake was estimated using the 2011 Dutch Food Composition Table^(^ ^27^ ^)^.

In order to assess differences in dietary quality between baseline and follow-up, a modification of the Dutch Healthy Diet index 2015 (DHD15-index) was calculated^(^ ^28^ ^)^. The DHD15-index estimates the level of adherence to the most recent 2015 Dutch dietary guidelines from the Dutch Health Council^(^ ^29^ ^)^. We were able to calculate twelve of the fifteen original components (see online supplementary material, Supplemental Table S1). For each component, a score between 0 and 10 was calculated. Consequently, the DHD15-index score in our study could range from 0 to 120 points, with higher scores indicating a healthier diet. We excluded the components for coffee and salt consumption, because the type of coffee (filtered or unfiltered) and salt consumption were not available in both FFQ. Two components needed to be adapted to our data. First, the wholegrain components originally had two components (both 5 points), one for total wholegrain product intake and one for the ratio between wholegrain and refined-grain products. Our follow-up questionnaire did not differentiate between types of cereals (wholegrain or not). Therefore, the wholegrain component was based on wholegrain bread only. Consumption equal to or above 90 g of wholegrain bread daily received the maximum score of 10 points, gradually decreasing to 0 points at a consumption of 0 g/d. Second, separate variables for red and processed meat were not available for the follow-up questionnaire, so these two components were combined. Consumption below 45 g of red and processed meats daily received the maximum score of 10 points, gradually decreasing to 0 points at consumption of 150 g/d or more.

Additionally, the food groups were classified by source category: animal-based, plant-based, beverages and miscellaneous. Animal-based foods included red and processed meat, poultry (shell)fish, eggs and dairy (including cheese). Plant-based foods contained potatoes, fries, bread and cereals, fruit, vegetables, vegetarian meat replacers, and nuts/seeds/nut paste. Beverages included coffee, tea (light) soft drinks, fruit and vegetable drinks, and alcoholic beverages. Miscellaneous contained savoury snacks, cakes/cookies, soups, sauces, oils and fats, and sweets. The twelve DHD15-index components can also be divided in these categories. Animal-based were the components dairy, (shell)fish, and red and processed meat with a combined maximum score of 30 points on the DHD15-index. Plant-based components were vegetables, fruit, wholegrain bread, legumes, and nuts/seeds/nut paste with a combined maximum score of 50 points. In the beverages category, the components tea, sweetened beverages and fruit juices, and alcoholic beverages were included with a maximum total score of 30 points. The last category, miscellaneous, included component for replacing butter and hard fats with oils and margarines for a maximum of 10 points.

### Environmental impact assessment

To estimate the GHG emissions associated with foods in the Netherlands, the methodology of life-cycle assessment was applied. The life-cycle assessment was performed using Life Cycle Inventory (LCI) data (Blonk Consultants, data set version 2016) from Agri-Footprint^(^ ^30^ ^,^ ^31^ ^)^. These LCI data were representative for the Dutch situation. These LCI data were used as input for life-cycle impact assessments using ReCiPe version 2008 and carried out by the Dutch National Institute for Public Health and the Environment (RIVM). The life-cycle impact assessments were cradle to plate and included all life-cycle stages from production, transport to preparation, and including waste/losses at all stages. The preparation of foods by consumers was based on the average cooking time for each product and an energy mix representative for the Dutch market. Food waste was included by using food-group-specific percentages for avoidable and unavoidable food losses at all stages of the life cycle. The time horizon for the effects of GHG emission calculations was 100 years and economic allocation was used for production processes that led to more than one food product. GHG emissions were expressed as kilograms of CO_2_-equivalents per kilogram of food prepared at plate (kg CO_2_-eq/kg). The environmental data used were previously presented by Van de Kamp *et al.* ^(^ ^31^ ^)^. The life-cycle assessment data were combined with the EPIC-NL FFQ data both at baseline and at follow-up to calculate daily GHG emissions associated with the usual diet in kilograms of CO_2_-equivalents per person per day (kg CO_2_-eq/person per d). Although improvements in production over time may most likely have decreased the environmental impact, we applied the same life-cycle assessment data to both the baseline and follow-up FFQ because then the observed differences in environmental impact are directly related to the dietary changes.

### Lifestyle and anthropometric variables

For the description of our research population at baseline, several lifestyle and anthropometric variables were measured. The study participants completed a standardized structured general questionnaire on the presence of chronic diseases, related potential risk factors and lifestyle factors. Blood pressure, weight and height were measured by trained staff according to standardised protocols^(^ ^17^ ^)^. BMI was calculated by dividing weight by height squared (kg/m^2^). Physical activity was assessed with a validated questionnaire^(^ ^32^ ^)^ and classified according to the Cambridge Physical Activity Index (CPAI) with imputed data for missing values (*n* 693)^(^ ^33^ ^)^. The CPAI was categorized into inactive, moderately inactive, moderately active and active. Smoking was operationalized as current, former and never smoker. Educational level was coded as low (lower vocational training or primary school), medium (intermediate vocational training or secondary school) or high (higher vocational training or university).

### Statistical analysis

All analyses were stratified by sex. First, the DHD15-index score was calculated. Second, the environmental impact of the diet at baseline and follow-up was calculated. We analysed the GHG emissions absolute (total) and relative per 4184 kJ (1000 kcal). Third, the differences in food group consumption and food sources over time were calculated. In order to get insight in the differences in consumption of food groups independently from differences in energy intake over time, consumption was standardized by energy intake (g/4184 kJ). Mean and sd values at baseline and follow-up were calculated for each indicator (the DHD15-index score, GHG emissions (kg CO_2_-eq/4184 kJ) and food groups (g/4184 kJ)). A paired-sample *t* test was used to test the observed differences for significance. A *P* value below 0·05 was considered statistically significant and all analyses were performed with the statistical software package SAS version 9.4.

## Results

Mean age at baseline was 51 years for women and 44 years for men in our cohort. At baseline, the majority of the participants were at least moderately physically active, moderately to highly educated, and most often either never smokers or former smokers (Table 1). Fifty-five per cent of men were overweight (BMI ≥ 25 kg/m^2^) or obese (BMI ≥ 30 kg/m^2^), whereas this was 39 % for women. At follow-up, both among men and women an increased prevalence of obesity (BMI ≥ 30 kg/m^2^) as well as underweight (BMI < 18 kg/m^2^) was observed. Men and women reported a decreased energy intake (–678 (95 % CI –4908, 3377) kJ/d (–162 (95 % CI –1173, 807) kcal/d) for men and –372 (95 % CI –3820, 3130) kJ/d (–89 (95 % CI –913, 748) kcal/d) for women) between baseline and follow-up (Table 2).

**Table 1:** Characteristics of the European Prospective Investigation into Cancer and Nutrition – Netherlands (EPIC-NL) study population

Characteristic	1993–1997				2015			
	Men (*n* 1844)		Women (*n* 7088)		Men (*n* 1844)		Women (*n* 7088)	
	%		*n*		%		*n*	
Age at baseline								
20–35 years	16·7	308	8·7	619	23·9	441	8·2	584
35–50 years	49·3	910	22·7	1612	7·2	132	4·1	293
50–65 years	33·9	626	64·4	4561	29·1	536	11·5	818
65–80 years	0·1	2	4·2	296	39·1	722	62·2	4408
>80 years	–	–	–	–	0·7	13	13·9	985
Education level								
Low	22·6	416	29·6	2096	NA			
Moderate	38·1	702	42·3	2998				
High	39·3	726	29·1	1994				
Physical activity								
Inactive	6·5	120	3·9	274	NA			
Moderately inactive	24·4	449	24·6	1741				
Moderately active	28·0	517	27·1	1926				
Active	41·1	758	44·4	3147				
Smoking status								
Yes	28·0	517	20·6	1455	12·2	165	7·3	445
No	35·1	645	43·2	3055	36·5	492	46·5	2864
Former	36·9	678*	36·2	2561†	51·3	692‡	46·3	2850§
BMI								
<18 kg/m^2^	0·3	5	0·7	51	15·1	279	11·0	780
18–25 kg/m^2^	44·7	824	60·6	4296	40·7	751	43·3	3071
25–30 kg/m^2^	46·2	853	30·8	2181	33·6	619	31·6	2238
>30 kg/m^2^	8·8	162	7·9	560	10·6	195	14·1	999

NA, not available.

* Missing: *n* 4.

† Missing: *n* 17.

‡ Missing: *n* 495.

§ Missing: *n* 929.

**Table 2:** Mean and sd baseline and follow-up food consumption and dietary greenhouse gas emissions in the European Prospective Investigation into Cancer and Nutrition – Netherlands (EPIC-NL) cohort

Food group	Men (*n *1844)								Women (*n* 7088)							
	g/4184 kJ (1000 kcal)				kg CO_2_-eq/4184 kJ (1000 kcal)				g/4184 kJ (1000 kcal)				kg CO_2_-eq/4184 kJ (1000 kcal)			
	Baseline		Follow-up		Baseline		Follow-up		Baseline		Follow-up		Baseline		Follow-up	
	Mean	sd	Mean	sd	Mean	sd	Mean	sd	Mean	sd	Mean	sd	Mean	sd	Mean	sd
Total energy intake (kJ/d)	10 109	2054	9431***	2414					7782	1774	7406***	2180				
Total energy intake (kcal/d)	2416	491	2254***	577					1860	424	1770***	521				
Animal-based foods																
(Shell)Fish	4·2	4·5	13·6***	14·9	0·04	0·04	0·13***	0·14	5·8	6·2	16·8***	19·1	0·05	0·06	0·16***	0·19
Red and processed meat	49·5	22·0	48·5	28·9	0·95	0·47	1·07***	0·76	42·7	22·3	42·2	29·2	0·88	0·51	0·97***	0·78
Poultry	5·6	5·8	12·5***	14·8	0·07	0·07	0·15***	0·18	6·3	6·7	13·0***	15·6	0·08	0·08	0·16***	0·19
Cheese	15·1	11·3	7·1***	7·6	0·15	0·11	0·07***	0·07	20·1	13·0	9·0***	9·5	0·20	0·13	0·09***	0·09
Low-fat dairy	92·6	82·2	96·6	95·9	0·17	0·15	0·19***	0·19	145·8	108·6	149·2*	120·0	0·28	0·20	0·30***	0·24
High-fat dairy	59·6	51·4	21·0***	33·0	0·13	0·11	0·04***	0·06	70·3	56·3	19·1***	33·7	0·16	0·13	0·04***	0·07
Eggs	7·2	5·7	10·3***	9·6	0·04	0·03	0·05***	0·05	8·0	6·3	12·4***	11·3	0·04	0·03	0·06***	0·06
Plant-based foods																
Fries	11·4	9·5	7·8***	11·2	0·04	0·03	0·03****	0·04	3·6	7·2	3·9*	8·5	0·01	0·03	0·01*	0·03
Potatoes	42·6	26·9	36·9***	27·7	0·03	0·02	0·05***	0·03	39·4	25·8	37·2***	28·4	0·03	0·02	0·04***	0·03
Low-fibre bread	15·9	19·1	25·8***	28·6	0·03	0·03	0·03	0·03	12·7	13·4	17·4***	24·2	0·02	0·02	0·02	0·03
High-fibre bread	58·2	30·8	38·2***	32·2	0·06	0·03	0·04****	0·03	54·0	24·5	39·0***	30·4	0·05	0·02	0·04***	0·03
Fruit	68·8	55·3	64·9**	57·0	0·06	0·05	0·06	0·06	125·9	83·3	104·8***	77·7	0·11	0·08	0·10***	0·08
Cereals and cereal products	30·5	24·4	34·8***	29·7	0·04	0·04	0·05***	0·05	24·6	21·2	30·5***	25·9	0·03	0·03	0·04***	0·04
Vegetables and legumes	54·0	23·7	63·1***	41·1	0·07	0·03	0·08***	0·06	81·4	35·5	83·4*	56·3	0·10	0·04	0·11***	0·08
Nuts, seeds and nut-paste	4·7	5·5	7·1***	7·8	0·01	0·01	0·02***	0·02	4·2	5·3	6·6***	8·1	0·01	0·01	0·02***	0·02
Vegetarian meat replacers and soya products	0·9	2·9	4·2***	19·1	0·001	0·005	0·01***	0·02	1·4	4·2	7·8***	31·4	0·002	0·01	0·01***	0·03
Beverages																
Coffee	273·7	167·9	212·5***	154·3	0·08	0·05	0·07***	0·05	294·9	180·5	234·3***	160·6	0·09	0·06	0·08***	0·06
Tea	94·8	123·8	120·1***	145·6	0·02	0·02	0·02***	0·03	207·4	188·4	255·9***	227·9	0·04	0·03	0·05***	0·04
Light soft drinks	13·1	27·8	34·8***	82·0	0·01	0·02	0·02***	0·05	16·3	32·1	21·1***	68·9	0·01	0·02	0·01***	0·04
Soft drinks, fruit and vegetable juices	59·8	54·5	65·1*	78·3	0·05	0·05	0·06**	0·06	66·2	60·1	65·6	85·7	0·06	0·06	0·06	0·08
Alcoholic beverages	127·2	132·7	128·3	129·0	0·13	0·13	0·15***	0·14	54·5	71·2	65·2***	82·1	0·08	0·09	0·10***	0·12
Miscellaneous																
Savoury snacks	10·4	9·1	8·9***	9·6	0·03	0·04	0·04***	0·05	6·0	5·9	5·9	7·7	0·02	0·03	0·03***	0·04
Cakes/cookies	11·4	9·0	14·8***	13·2	0·02	0·02	0·04***	0·03	17·2	11·5	17·3	13·8	0·03	0·02	0·04***	0·03
Soups	33·5	36·4	23·9***	30·6	0·11	0·12	0·04***	0·06	36·8	39·5	27·2***	37·8	0·13	0·13	0·05***	0·07
Sweets	18·5	12·5	19·5*	16·6	0·02	0·02	0·05***	0·06	15·9	11·9	19·0***	16·2	0·02	0·02	0·05***	0·05
Savoury sauces	9·9	8·3	5·7***	5·9	0·02	0·01	0·01***	0·01	9·7	8·0	5·9***	6·8	0·01	0·01	0·01***	0·01
Oils, diet margarine, liquid cooking fats	5·0	4·8	11·7***	11·1	0·01	0·01	0·03***	0·02	5·1	4·2	9·7***	9·8	0·01	0·01	0·02***	0·02
Butter and solid cooking fats	7·3	5·7	2·3***	5·3	0·04	0·04	0·02***	0·06	6·6	5·1	3·2***	5·9	0·04	0·04	0·03***	0·07

Significance level of paired *t*-test: *P < 0·05, **P < 0·001, ***P < 0·0001.

**Fig. 1:**
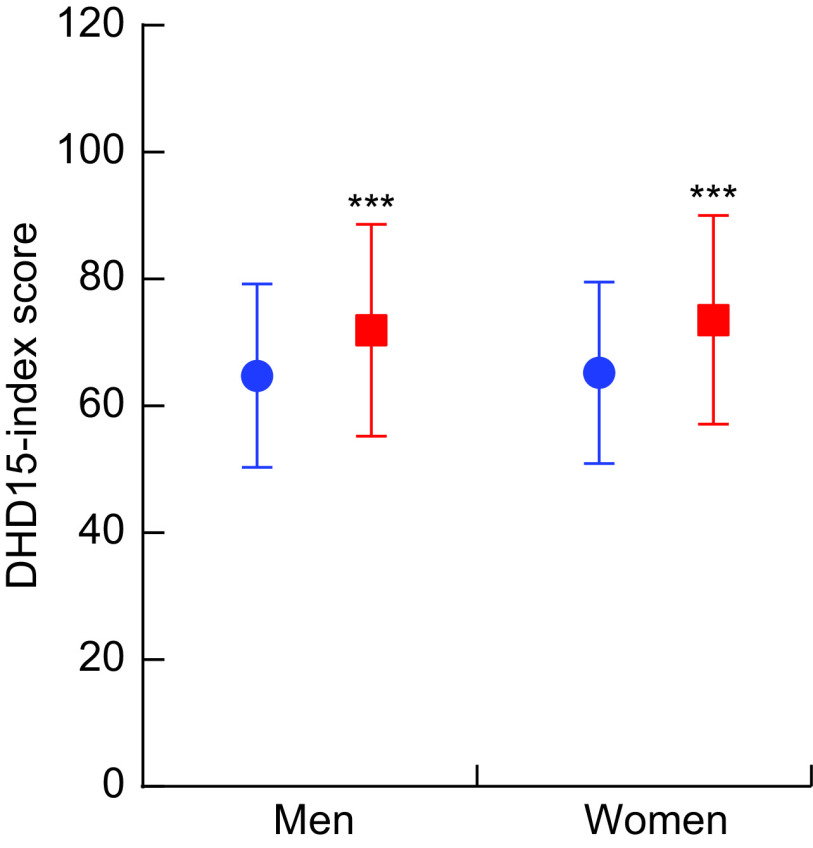
Scores on the Dutch Healthy Diet index 2015 (DHD15-index) at baseline (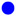) and follow-up (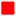) in the European Prospective Investigation into Cancer and Nutrition – Netherlands (EPIC-NL) cohort. Values are means with their sd represented by vertical bars. Significance level of paired *t* test: ***P < 0·0001

For both men and women, a statistically significant increase of about 12 % in the DHD15-index score was observed at follow-up compared with baseline, indicating an improved dietary quality between 2015 and 1993–1997 (Fig. 1). At follow-up, on average men had a score of 71·9 (95 % CI 41·9; 97·8) points and women a score of 73·6 (95 % CI 45·3; 99·8) points. The increase in DHD15-index score was mostly due to a higher consumption of (shell)fish and nuts/seeds/nut paste (see online supplementary material, Supplemental Table S1). The two component scores of the DHD15-index that had the largest decreases in score were wholegrain bread and vegetables.

**Fig. 2:**
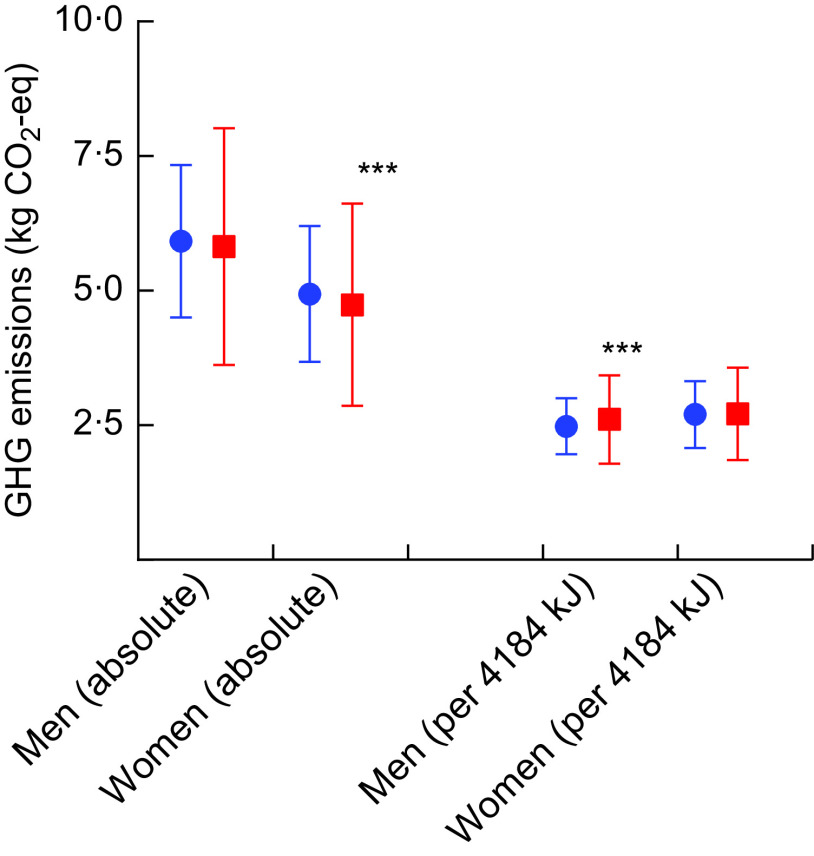
Greenhouse gas (GHG) emissions, absolute and per 4184 kJ (1000 kcal), at baseline (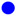) and follow-up (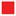) in the European Prospective Investigation into Cancer and Nutrition – Netherlands (EPIC-NL) cohort. Values are means with their sd represented by vertical bars. Significance level of paired *t* test: ***P < 0·0001

Total daily GHG emissions of the diet were respectively 2 and 4 % lower in men (5·82 *v.* 5·92 kg CO_2_-eq/person per d, *P* = 0·052) and women (4·74 *v.* 4·94 kg CO_2_-eq/person per d, *P* < 0·0001) in 2015 compared with 1993–1997 (Fig. 2). After adjusting for energy intake, diets of men were associated with a statistically significant 5 % higher GHG emissions in 2015 compared with diets at baseline when expressed per 4184 kJ (1000 kcal; 2·61 *v.* 2·48 kg CO_2_-eq/4184 kJ per d, *P* < 0·0001), while diets of women had similar relative dietary GHG emissions (*P* = 0·3930).

**Fig. 3:**
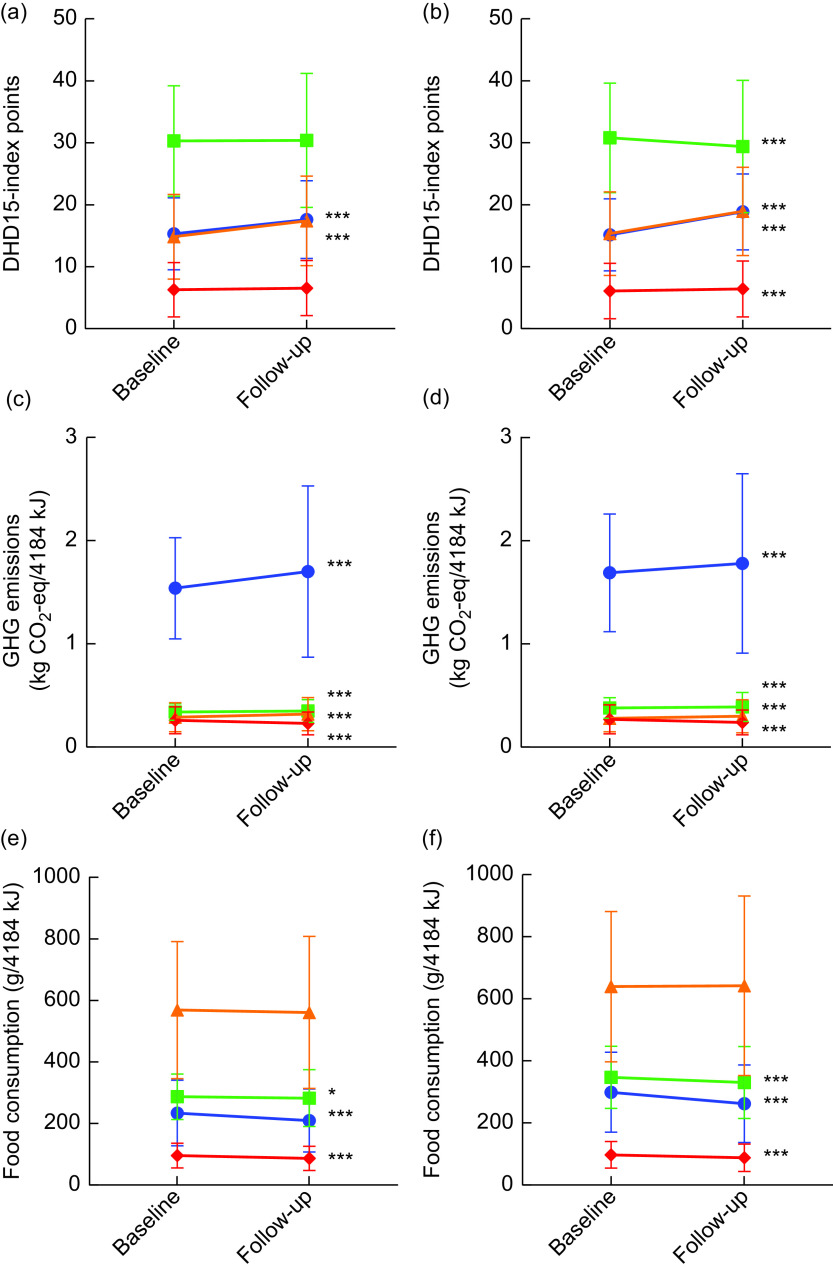
(a, b) Points on the Dutch Healthy Diet 2015 index (DHD-15 index), (c, d) greenhouse gas (GHG) emissions per 4184 kJ (1000 kcal) and (e, f) food consumption per 4184 kJ, at baseline and follow-up, by sex (a, c, e, men; b, d, f, women) and per food type† (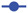, animal-based foods; 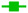, plant-based foods; 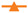, beverages; 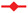, miscellaneous), in the European Prospective Investigation into Cancer and Nutrition – Netherlands (EPIC-NL) cohort. Values are means with their sd represented by vertical bars. Significance level of paired *t* test: *P < 0·05, ***P < 0·0001. †Animal-based foods include red and processed meat, poultry (shell)fish, eggs and dairy. Plant-based foods contain potatoes, fries, bread and cereals, fruit, vegetables, vegetarian meat replacers, nuts, seeds and nut paste. Beverages include coffee, tea (light) soft drinks, fruit and vegetable drinks, and alcoholic beverages. Miscellaneous contains savoury snacks, cakes/cookies, soups, sauces, oils and fats, and sweets. Animal-based foods could score a potential 30 points on the DHD15-index, plant-based foods 50 points, beverages 30 points and miscellaneous 10 points

Animal-based foods consumption was healthier according to the DHD15-index score for animal-based foods but less environmentally friendly according to the GHG emissions per 4184 kJ (1000 kcal). A potential 30 points could be scored on the DHD15-index for animal-based foods, on which both men (15·4–17·6 points) and women (15·5–18·8 points) had increased scores (Fig. 3(a) and (b)). The associated GHG emissions of the animal-based foods statistically significantly increased from 1·54 to 1·70 and from 1·69 to 1·78 kg CO_2_-eq/4184 kJ in men and women, respectively (Fig. 3(c) and (d)), mainly because of the higher (shell)fish, poultry, and type of red meat consumption (Table 2). Total red and processed meat consumption per 4184 kJ (48·5 g/4184 kJ at follow-up for men and 42·2 g/4184 kJ for women) was similar, thus meats with relative higher GHG emissions were more often consumed.

For the plant-based foods, at similar energy intakes, healthiness of diets and GHG emissions was almost similar. A potential 50 points could be scored on the DHD15-index for plant-based foods, on which men scored similar but women decreased their score. The GHG emissions of total plant-based foods increased only slightly but statistically significantly in men and women. Within the plant-based foods, an increased consumption (g/4184 kJ (1000 kcal)) of low-fibre bread, cereals, vegetables and legumes, nuts/seeds/nut paste and vegetarian meat replacers was observed whereas the consumption of potatoes, fruit and fries (men only) decreased.

The difference in consumption of miscellaneous foods resulted in slightly lower relative GHG emissions and (men and women) and higher DHD15-index scores (only women). Miscellaneous food groups of which the consumption increased at follow-up were cakes/cookies, sweets, and oils, diet margarines and liquid cooking fats, whereas a decreased consumption of savoury snacks and sauces, soups, and butter and solid cooking fats was reported. Within the category beverages, our participants reported a decreased coffee consumption (~60 g/4184 kJ (1000 kcal) less) and increased consumption of tea, light soft drinks, soft drinks (men only) and alcoholic beverages (women only; Table 2), resulting in somewhat higher GHG emissions/4184 kJ (1000 kcal) but also higher DHD15-index scores.

## Discussion

Overall, energy intake decreased by 678 kJ/d (162 kcal/d) for men and 372 kJ/d (89 kcal/d) for women. The observed dietary changes resulted in a healthier diet, as measured by the DHD15-index score. The average score was 12 % higher at follow-up, mainly due to increased consumption of fish and nuts/seeds/nut paste. Absolute GHG emissions of the diet were slightly lower in women (−4 %) only. Expressed per 4184 kJ (1000 kcal), the GHG emissions of the diet were higher in men (+5 %, *P* < 0·0001) and remained similar in women (+0·4 %), indicating no mitigation of GHG emissions. Higher consumption of (shell)fish and poultry was not yet at the expense of red and processed meats.

The Dutch national food consumption surveys between 1987 and 2010 showed similar meat, cheese, dairy and bread consumption. Rice, pasta and non-alcoholic beverages consumption increased and potatoes, fruit and vegetable consumption decreased^(^ ^34^ ^)^. In 2012, the consumption of potatoes was even lower, while similar amounts of vegetables were consumed and slightly less cheese, dairy and meat^(^ ^35^ ^)^. Comparing a similar time frame (between 1987 and 2010), red and processed meat consumption remained similar in our cohort as well, while the type of red meat changed from beef to pork. Total meat and fish consumption increased because of the increased poultry and fish consumption. On the other hand and not in line with results of the Dutch national food consumption survey^(^ ^34^ ^)^, we observed an increased cereal and vegetables consumption as well as decreased bread (especially high-fibre bread) and cheese consumption.

For all of the differences noted in our analyses, we did not investigate whether it is an effect of the ageing of our cohort or a difference between time points. Especially the reduction in energy intake would likely be an ageing effect. A new study could also stratify the data by age group to compare both ageing as well as time effects simultaneously. This was, however, beyond the scope of our current study. Since most of the observed changes in diet in our cohort were comparable to other Dutch cross-sectional studies over time^(^ ^16^ ^,^ ^34^ ^)^, we hypothesize that they are a time effect and not necessarily an ageing effect.

A Canadian study identified 10-year dietary changes and its related GHG emissions between two cross-sectional surveys (in 2004 and 2015) using two 24 h recalls^(^ ^35^ ^)^. Overall, total amount of energy consumed was 20 % lower in 2015 compared with 2004. The authorobservedalower consumption of beef (−29 %), dairy (−22 %) pork (−11 %), sausages (−31 %), and fruit and vegetables (−16 %). In addition, a higher consumption of poultry (+18 %), fish (+11 %) and nuts (+43 %) was observed. The total dietary GHG emissions in the Canadian study were 28 % lower in 2015 than in 2004, which could be attributed mainly to the lower energy intake and the lower beef consumption. Although the observed differences in GHG emissions and consumption of food groups were in the same or similar direction as in our study, much larger differences were found, especially for the consumption of red and processed meats. Similar to our study, consumption and GHG emissions due to poultry and fish consumption increased over time.

Some changes in the diet might be linked to increased awareness among consumers of what constitutes a healthy diet. Over the years, the Dutch Health Council and the Netherlands Nutrition Centre have been presenting guidelines for a healthy diet. In 2006, they highlighted the benefits of a higher consumption of fish, vegetables and fruits, and risks of a high consumption of solid fats, alcohol and salt^(^ ^36^ ^)^. The updated guidelines, issued in 2015 after our measurements, included more food groups and had a focus on a plant-based instead of an animal-based diet (more vegetables, fruits and nuts, less red meat, no processed meat, dairy within a range)^(^ ^29^ ^)^. The observed increased consumption of (shell)fish, nuts/seeds/nut paste, legumes and meat replacers, and the decreased consumption of fries and snacks in our population are in line with these new recommendations. The consumption of (red and processed) meats and dairy within a range needs further attention and additionally may lead to diets with lower GHG emissions.

Our results showed improvements in the dietary quality of the diets, as measured by the DHD15-index, but not more environmentally friendly diets, as measured via GHG emissions. Considering the minimal reduction in absolute consumption of red and processed meat, the higher GHG emissions from animal-based food consumption (meat, poultry, fish, dairy (including cheese) combined) and the below recommendation consumption of fruit and vegetables (290 *v.* 400 g/d), these parts are key in new food policies and communication targeting both health as well as environmental aspects. The observed increase in DHD15-index score (men scored 7 points higher and women 8·5) is relevant for public health. In a previous study with baseline data of EPIC-NL we observed that per 1 sd increase in the DHD15-index score (16 points), the overall risk of mortality was 12 (95 % CI 5, 18) % lower in men and 8 (95 % CI 4, 12) % lower in women^(^ ^37^ ^)^.

The applied FFQ were validated against 24 h recalls but showed some differences in measurement error for several food groups^(^ ^23^ ^,^ ^24^ ^,^ ^26^ ^)^. The validation studies indicate similar correlations between both FFQ and 24 h recalls for meat, dairy and fish consumption. A higher correlation was observed for potatoes (0·58 *v.* 0·28), cheese (0·64 *v.* 0·16), sweets (0·78 *v.* 0·38) and cakes/cookies (0·56 *v.* 0·33) for the baseline compared with the follow-up FFQ. The correlation between vegetable consumption and 24 h recalls was higher in the follow-up FFQ (0·52 *v.* 0·38). Therefore, the observed differences between baseline and follow-up for these food groups must be interpreted with caution since the measurement error is larger when the correlation coefficient is lower. The reduction in cheese consumption (50 % less), for example, might be due to the large difference in correlation error of the FFQ to the 24 h recalls (baseline: 0·64 *v.* follow-up: 0·16). Furthermore, FFQ are designed to rank individuals and not to calculate absolute food consumption or energy intake. Although most observed changes in diet in our cohort were comparable to other Dutch cross-sectional studies based on 24 h recalls^(^ ^16^ ^,^ ^34^ ^)^, the absolute intakes should be interpreted carefully. The food composition tables used in our study have changed with regard to the calculation of energy^(^ ^25^ ^,^ ^27^ ^)^. Fibre content was used for energy calculations (8·4 kJ/g (2 kcal/g)) only in the table of 2011. Since we used energy-adjusted consumption (g/4184 kJ (1000 kcal)) in our analyses, we will slightly overestimate the g/1000 g of all food groups and the kg GHG emissions/4184 kJ in 1996 compared with 2015. The recommended amount of fibre consumption in the Netherlands is 40 g/d^(^ ^38^ ^)^, so this would add approximately 335 kJ/d (80 kcal/d) to the mean energy intake at baseline. For example, the red meat consumption in men would then be estimated as 47·9 g/4184 kJ instead of 49·5 g/4184 kJ, decreasing the already non-significant difference between baseline and follow-up. Since most Dutch people do not meet the recommendation for fibre (median habitual consumption 20 g/d^(^ ^39^ ^)^), this actual difference between the with and without fibre calculation at baseline will be even smaller. The number of total food items per FFQ also differ, 178 in the baseline questionnaire and 160 at follow-up, but the used food groups were similar. Excluding the several sub-questions on brands present in the baseline FFQ, both FFQ had a similar total number of foods. In addition, by aggregating food items to overarching food groups in our study, potential differences in specific food items within food groups likely have not played a major role.

Despite the time span of 20 years between the two FFQ, we applied the same data on GHG emissions of foods available in the Netherlands. Consequently, differences in the GHG emissions between the two time points can be directly related to differences in dietary consumption and not to possible changes in production methods or efficiency gains in certain processes. Because of these technological improvements in production over time, we are likely underestimating the GHG emissions at baseline, since the GHG emissions were based on the 2016 data. In our study, the environmental impact is an average for foods available in the Netherlands, such as imported and domestic foods. For example, for tomatoes a percentage is grown in greenhouses in the Netherlands and a percentage is field grown in Spain and imported. Because the FFQ does not provide information on country of origin and production method, such variations in GHG emissions could not be considered. The small differences in the GHG emissions associated with the observed changes in the diet should therefore be interpreted cautiously. In addition, food production not only affects GHG emissions, but also eutrophication, water use and acidification. However, data on GHG emissions are available most and are therefore used by us, and other recent studies^(^ ^40^ ^–^ ^44^ ^)^.

## Conclusion

In conclusion, the present Dutch cohort analyses showed more healthy diets without mitigated GHG emissions over a 20-year period, at similar energy intakes. Higher consumption of (shell)fish and poultry was not yet at the expense of red and processed meats. Lower consumption of animal-based foods is needed to achieve healthier as well as environmentally friendly diets.

## Supplementary material

For supplementary material accompanying this paper visit https://doi.org/10.1017/S1368980019001824. click here to view supplementary material
