# Supplementary material for: Are our diets getting healthier and more sustainable? Insights from the European Prospective Investigation into Cancer and Nutrition – Netherlands (EPIC-NL) cohort
Source: Public Health Nutr. 2019 Jul 31;22(16):2931–40. doi: 10.1017/S1368980019001824 (PMC6792144; doi:10.1017/S1368980019001824)
Supplement: Supplementary file 1 [file S1368980019001824sup.zip › S1368980019001824sup001.docx]

**Supplemental figure 1.** Flow chart of participants in the present study.
