# Supplementary material for: Are our diets getting healthier and more sustainable? Insights from the European Prospective Investigation into Cancer and Nutrition – Netherlands (EPIC-NL) cohort
Source: Public Health Nutr. 2019 Jul 31;22(16):2931–40. doi: 10.1017/S1368980019001824 (PMC6792144; doi:10.1017/S1368980019001824)
Supplement: Supplementary file 1 [file S1368980019001824sup.zip › S1368980019001824sup002.docx]

**Supplemental table 1.** Mean score and standard deviation on the subcomponents of the Dutch Healthy Diet index 2015 (DHD15-index) at baseline and follow-up stratified by sex^a^.

| **Food groups** | **Men** (n=1,844) | | | |  | **Women** (n=7,088) | | | |
| --- | --- | --- | --- | --- | --- | --- | --- | --- | --- |
|  | **Baseline** | | **Follow-up** | |  | **Baseline** | | **Follow-up** | |
| Vegetables | 6.4 | 2.4 | 6.0*** | 3.1 |  | 6.4 | 2.3 | 5.8*** | 3.1 |
| Fruit | 6.4 | 3.2 | 6.2 | 3.7 |  | 6.6 | 3.2 | 6.7* | 3.6 |
| Wholegrain bread | 7.4 | 3.5 | 6.5*** | 3.9 |  | 7.6 | 3.5 | 6.2*** | 3.9 |
| Legumes | 6.0 | 3.9 | 6.5 | 4.5 |  | 6.2 | 3.8 | 6.7*** | 4.5 |
| Nuts/seeds/nut paste | 4.0 | 3.6 | 5.7*** | 4.0 |  | 3.9 | 3.6 | 5.0*** | 4.1 |
| Dairy products | 5.8 | 3.6 | 5.7 | 3.4 |  | 5.8 | 3.6 | 6.3*** | 3.4 |
| Fish | 4.0 | 2.6 | 6.3*** | 3.6 |  | 3.8 | 2.6 | 6.1*** | 3.7 |
| Tea | 5.3 | 3.8 | 5.4 | 4.0 |  | 5.2 | 3.7 | 6.3*** | 3.9 |
| Replace butter and hard fats with margarines and oils | 6.3 | 4.4 | 6.5 | 4.5 |  | 6.1 | 4.5 | 6.4*** | 4.5 |
| Red meat and processed meat | 5.9 | 3.6 | 5.6*** | 3.9 |  | 5.9 | 3.5 | 6.4*** | 3.7 |
| Sweetened beverages and fruit juices | 4.6 | 3.4 | 5.8*** | 3.9 |  | 4.8 | 3.4 | 6.1*** | 3.8 |
| Alcohol | 6.4 | 4.2 | 6.3 | 4.3 |  | 6.4 | 4.2 | 6.6 | 4.3 |

a: Significance level paired t-test *<0.05, **<0.001, and ***<0.0001.
